# Assessment of Wastewater Treatment Plant Upgrading with MBR Implementation

**DOI:** 10.3390/membranes13080746

**Published:** 2023-08-21

**Authors:** Nikolay Makisha

**Affiliations:** Research and Education Centre “Water Supply and Wastewater Treatment”, Moscow State University of Civil Engineering, 26, Yaroslaskoye Highway, 129337 Moscow, Russia; makishana@mgsu.ru

**Keywords:** wastewater treatment, membrane bioreactor, modernization, activated sludge reactor, cost analysis

## Abstract

Modernization of wastewater treatment plants is usually caused by their significant wear and changes in the flow rate and concentration of pollutants. If there is no initial data on the flow or pollution, their determination by calculation is required, which may lead to an increase in concentration. Within the study, the modernization of treatment facilities was estimated under conditions of reduced flow and increased pollution concentration. Calculations were carried out both manually and using the CapdetWorks software package. The focus was on secondary treatment facilities as the main element of the municipal wastewater treatment plant within their upgrade from only organic pollutants removal (plug–flow reactor) to removal of both organic pollutants and nutrients (technology of the University of Cape Town). The calculations of tank volumes have shown that the concentration of pollutants has a much greater impact on them than the change in flow, especially when improvement in the treatment quality is required. The study revealed that membrane sludge separation allows tanks to be reduced in volume by 1.5–2.5 times (depending on the value of mixed liquor suspended solids) in comparison with gravity separation, which means smaller capital costs. However, membrane application requires significant energy costs for membrane aeration. For the initial data of the study, the specific energy costs for aeration before the upgrade, after the upgrade (gravity separation), and after the upgrade (membrane separation) were 0.12 kWh/m^3^, 0.235 kWh/m^3^, and 0.3 kWh/m^3^, respectively. If the membrane lifetime is 10 years, membrane costs were determined to be 10–15% of the energy costs for aeration.

## 1. Introduction

According to the statistics, the total number of centralized wastewater disposal systems (CWWDS) in the Russian Federation is about 9300. There is no exact information about the number of wastewater treatment plants (WWTPs), but nominally, it should be close to the number of CWWDS, i.e., about 9000. With a design flow of approximately 58 million m^3^/day, the average design flow of one CWWDS is about 6450 m^3^/day, and the average actual flow is about 2900 m^3^/day [1,2].

However, significant underloading of WWTPs in practice does not always mean easy operating conditions and an available reserve of capacity [3]. A significant part of WWTPs in rural settlements and small towns is in poor condition, and some may be completely out of order. At many larger WWTPs, some of the treatment lines have been decommissioned due to unsatisfactory technical conditions. In addition, the design performance in the vast majority of situations allows the removal of organic pollutants only, while nutrient removal may require about 2–3 times higher hydraulic retention time (HRT) [4,5]. It is also essential that the reduction of wastewater quantity, which took place in the last 25 years, did not mean a proportional reduction in the pollution load, which largely determines the HRT in the WWTP [6].

Thus, the average load of 50% of the design capacity only shows some probability of upgrading these WWTPs with the implementation of new technologies without the construction of additional volumes of tanks [7,8].

Before the study, the statistics of the operation of 200 WWTPs were analyzed with the following distribution according to their design performance:

>300 k m^3^/day—20 WWTP;

100–300 k m^3^/day—30 WWTP;

<100 k m^3^/day—150 WWTP.

The majority of WWTPs in all three ranges of capacity were developed between 1970 and 1985. Until 1990, large- and medium-sized WWTPs were completed. Depending on the capacity, only 6% to 13% of the facilities were erected after 2000 [3,9,10].

Until 1990, the main task was to complete the existing WWTP facilities or build additional blocks. After the year 2000, new WWTPs were built to apply up-to-date methods of removal nitrogen (N) or nitrogen and phosphorus (P), both called nutrients. The modernization of WWTPs, which was carried out from 2000 to 2020, was also aimed at technological improvement. Despite the substantial percentage of objects undergoing modernization in two of the three groups, upgrading measures mostly involved the rejection of chlorination in favor of ultraviolet (UV) treatment and the switch to mechanical dewatering [11,12,13].

The share of facilities that use N-removal technology (or the removal of N and P) does not exceed 10% [14]. The daily specific rate of wastewater production (SRWP, L per capita) reveals that WWTP capacity may vary in a wide range (Table 1).

Table 1 shows that a decrease in WWTP capacity leads to a significant extension of the SRWP range. For the first group of treatment facilities, this difference between the minimum and maximum values of the SRWP is 2.5 times; for the second, five times; and for the third, nine times. It is worth saying that values of less than 100 L per capita correspond to incomplete drainage and/or a partial absence of CWWDS. In general, for WWTPs with a capacity of 100–300 thousand m3/day and especially less than 100 thousand m3/day, there is a reserve for reducing water consumption (and, accordingly, inflow to the WWTP) by taking water-saving measures. These facilities were also analyzed from the point of view of the main pollutants. The distribution of wastewater pollution by the total suspended solids (TSS) and biological oxygen demand (BOD5) is presented in Table 2, and the distribution of wastewater pollution by N and P is presented in Table 3.

Table 2 shows that regardless of the WWTP capacity, wastewater is mainly medium-concentrated in the BOD_5_ and TSS. The distribution of nutrients shows that the WWTP in the first range is characterized by an average concentration (2/3 of all WWTPs). In addition, for the third range, the distribution is almost uniform. This suggests that with a decrease in capacity and a larger range of the SRWP, the influence of concentrated effluents increases. The SRWP decrease means that 25–35% of the WWTP with a capacity of more than 100 k m^3^/day is receiving wastewater, which should be assessed as highly concentrated in the conditions of the Russian Federation [16]. In Western Europe, especially Germany, with the SRWP value in many localities below 80 L per capita, the concept of highly concentrated wastewater is significantly different: it is characterized by at least twice the pollution values for the TSS and BOD_5_. This fact has a significant impact when using treatment technologies developed for Western Europe in the Russian Federation [17,18].

Despite the decrease in specific wastewater disposal, about 1/3 of all WWTPs with a capacity of less than 300 k m^3^/day receive low-concentrated wastewater, including nitrogen and phosphorus [19].

## 2. Materials and Methods

The modernization of WWTPs is an extremely urgent task whose solution always takes place under various limitations. As a rule, these are financial limitations, area limitations, or limitations in available equipment or technologies [20].

**Figure 1:**
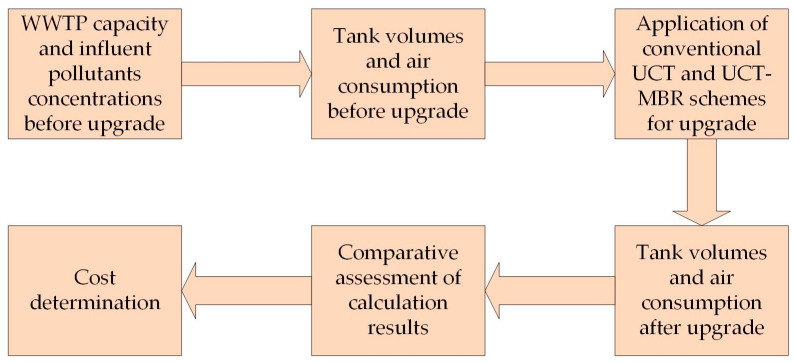
Scheme of the research.

In this research, the calculated justification of modernization was considered using the example of a virtual WWTP with typical characteristics. However, the sequence of necessary calculations, which usually precede the modernization of real WWTPs, corresponded to generally accepted practice. The scheme of the research is shown in Figure 1.

Currently, various WWTP calculation methods are used, and these methods have become significantly more complicated due to the increase in the number of variables. Stepanov [21] describes one of the techniques used in WWTPs in the Russian Federation and some neighboring countries.

In addition, there are various automated tools, such as GPS-X, BioWin, Design2Treat, and Belebungsexpert. Automated calculation tools, which, according to the developers, should facilitate the work of users, may not always have a user-friendly interface, which can certainly complicate and lengthen the calculation process.

It is especially important to obtain correct results, which could be verified manually if necessary. Thus, it is extremely important that manual calculation (MC) and software systems demonstrate a high convergence of results with a good degree of approximation.

However, the calculation of the technological parameters of the WWTP is a relatively small part of the entire wastewater treatment plant project. At the same time, such a calculation will be the starting point for many other calculations also related to the life cycle of such objects. That is, the correct calculation of technological parameters and the selection of tanks, facilities, equipment, and other elements of the station will allow for the determination of the capital costs of their construction as accurately as possible. In addition, it will be easier to predict operating costs, which, as is known in the context of the entire cycle, can significantly exceed capital costs.

With MC, this process can be time-consuming, and the use of software systems can significantly speed up this process. At the same time, it will be easier to link a large array of interdependent variables.

In previous studies, the use of CapdetWorks 4.0 software (CDW) was analyzed, but the issue of convergence with MC was not considered [22,23].

When calculating treatment facilities, the selection of preliminary treatment facilities is relatively simple, even in manual mode. The most difficult is the selection of secondary treatment facilities (ST), which are essentially the main element of any urban (municipal) WWTP. Correct calculation implies taking into account a wide range of variable parameters, whose correlation can be quite complicated [24].

As a rule, activated sludge reactors (ASRs) of various technical and technological designs are used for municipal wastewater treatment in 90–95% of cases. These facilities determine the quality of treatment according to the main parameters (removal of carbon and nutrients), contribute to the total energy consumption of the WWTP (up to 60% of the costs), and affect the amount of sludge generated and, accordingly, the costs of its treatment and disposal. Thus, in the current study, the main focus will be on secondary treatment facilities [2,5,10].

With full-fledged initial data, it is possible to start work on the design justification for the WWTP modernization. As a rule, three typical reasons arise for modernization: wear (obsolescence) of existing structures, changes in wastewater flow, and changes in the concentration of pollutants [25].

Wear on structures is usually expressed in the partial or complete destruction of tanks and system elements. At the same time, various factors affect the wear, which is why the destruction process occurs unevenly. Since this study is of a simulation nature, the assessment of wear will not be given in it, and the most interesting is the change in the quantitative indicators of treatment facilities [10,11].

Changes in the flow of wastewater and, as a consequence, the performance of treatment facilities can occur for various reasons. Recently, there has been a global trend toward optimizing water consumption, which, among other things, is expressed in reducing the amount of water used. In the residential area, this is mostly caused by the use of various water-saving household devices [26].

A significant change in the concentration of pollutants is a multifactorial process. It can be caused by a SRWP change that leads to an altered ratio between wastewater and dissolved pollutants. In addition, the change may occur because of the appearance of a new source of pollution or its exclusion from the city’s drainage system [27].

## 3. Results

The majority of municipal WWTPs were built between the 1970s and 1990s, and their design method was focused on the removal of only organic pollutants (carbon) and suspended solids [16,22]. Upgrades are usually based on existing information about treatment facilities. If any information is missing, it is permissible to obtain the missing data by calculation. In practice, similar calculations are required for many objects.

**Figure 2:**
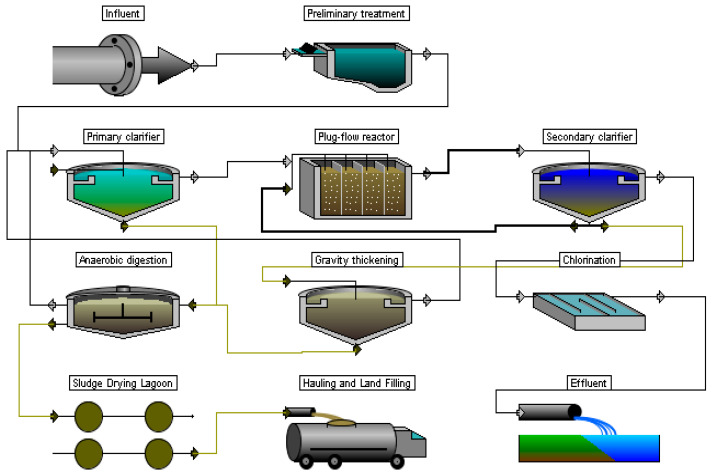
Sequence of treatment before modernization.

As part of this study, it is planned to carry out a design justification for the modernization of WWTPs, and the initial data will be determined by calculation. First, the initial and common technological scheme of treatment (SoT) was chosen, which was used to remove organic pollutants and suspended solids (Figure 2). Next, it was necessary to carry out its enlarged calculation to obtain a set of initial data for this study.

For an approximate flow determination, statistical information will be used. As of the beginning of 2020, in Russia, there were 1117 cities of various sizes, and 793 (70% of the total number) of them had a population of up to 50,000 people [28]. For the calculation, a population of 50,000 people will be used as the base. For structures that were designed and erected between the 1970s and 1990s, as a rule, the daily rate of water consumption (and drainage, as a consequence) was taken in the range of 350 to 500 L per capita. Thus, the average daily consumption of sewage treatment plants for a city with a population of 50,000 people, depending on the norm, will be approximately 17,500–25,000 m^3^/day. The minimum flow of 17,500 m^3^/day will be taken. The preliminary selection itself was carried out based on the calculation methodology in force at that time [29].

### 3.1. Analysis of the WWTP before Modernization

As a rule, the information provided in this subsection is already available at the beginning of the WWTP upgrade. Information about the dimensions of the tanks and the characteristics of the equipment can usually be obtained by analyzing the documentation or by visual inspection of the object. However, as mentioned above, often the necessary data are only partially available, so they have to be obtained by calculation, which will be demonstrated within the research [25].

The primary indicators of pollutant concentrations based on the specific amount of pollutants per capita were used as the initial data (Table 4). This indicator is particularly useful in design when reliable actual wastewater statistics are unavailable and only the population is known. The calculation was made with Equation (1). The initial values are generally representative of the average quality of municipal wastewater entering treatment plants.

(1) $$C_{i}=\frac{1000\times A_{PC}}{Q_{PC}}$$

where *A_PC_* is the daily amount of pollutants per capita; *Q_PC_* is the specific rate of wastewater production [L per capita day^−1^], which was accepted as 350 L per capita day^−1^ for the calculation.

At the pre-treatment stage, a standard solution with screens and sand traps is used. This is followed by the stage of primary sedimentation (clarification) of wastewater. The primary clarifier is usually rectangular or cylindrical in shape, but for our study, the choice of design is not fundamentally important. It should be noted that during primary sedimentation, key pollution indicators decrease: the TSS are usually up to 100–150 mg/L and the BOD_5_ by about 20%. As a result, after the primary settling and before the secondary treatment, the TSS = 112 mg/L (a clarification effect of 40%), and the BOD_5_ = 171 mg/L. The standard ASR in the form of a plug–flow reactor (PFR) was the most frequent option as an ST structure, which was a rectangular tank divided into corridors by partition walls. This facility was quite suitable to remove primarily organic pollutants, that is, carbon compounds, to a level equivalent to a BOD_5_ of 10–15 mg/L. The content of nutrients (N and P) in treated wastewater was not regulated in the Russian Federation until early 2000, so no special conditions were created for their removal in the PFR. In the calculations, the effect of nutrient concentration reduction was estimated at no more than 5% and, therefore, not taken into account. For real objects, the removal of nutrients from wastewater was also variable. In addition, so-called activated sludge regeneration was often used in the PFR, for which one or two corridors of the PFR tank were used.

In conventional ASRs, the separation of mixed liquor into treated water and activated sludge takes place in a secondary clarifier (SC). After this facility, the concentration of the TSS should not normally exceed 10–15 mg/L. Further, mandatory disinfection of wastewater was carried out, as a rule, with the help of chlorine compounds (chlorine gas and later sodium hypochlorite). The method of disinfection using sodium hypochlorite is still quite widespread.

As standard solutions for sludge treatment, gravity compaction structures and stabilization or fermentation, as well as dehydration, were used in various combinations. Using sludge-drying lagoons for dehydration was one of the most widespread solutions.

Since ST facilities play a key role in the treatment process, the main parameters of their operation were considered. For ASRs, the activated sludge parameters ensure the efficiency of treatment. The sludge concentration in the ASR or the mixed liquor suspended solids (MLSS) value in the range of 2–3 g/L was typically used in the PFR of similar solutions, while the sludge index was usually 80–120 mL/g. The practice of sewage treatment plants shows that the concentration of MLSS = 3 g/L, which is the most typical for the PFR, and this value will be taken as a reference [9].

**Figure 3:**
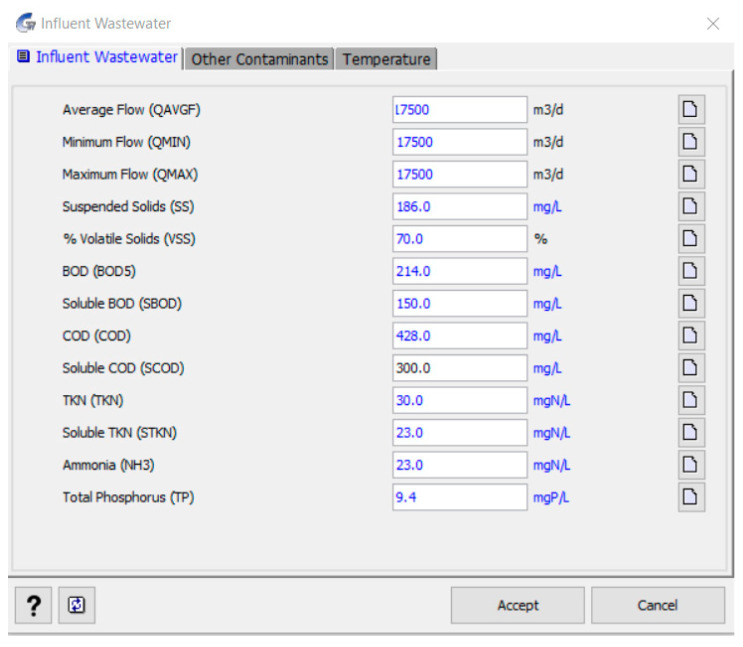
Input parameters of the CapdetWorks interface.

Based on these values, the oxidation rate, hydraulic retention time (HRT), and volume of the tanks can be calculated. The results of the calculation of the structures are presented in Table 5, while these calculations can be performed both manually by the method [29] and in the CapdetWorks environment [30]. The software constructs each unit process in a specified process layout based on the influent characteristics and then estimates the design cost. The two-step procedure allowed for the inspection of the generated design and, if necessary, modification with the program’s design override features. Typical design defaults have been used for each unit process to offer usable computed designs and make the program easier to use for planners who need planning-level cost estimates for a new facility or an upgrade to an existing facility. The input parameter interface is shown in Figure 3.

The wastewater temperature is taken in accordance with [31] and is equal to 14 and 22 for winter and summer, respectively.

Table 5 shows the main characteristics of the WWTPs that help to estimate the treatment efficiency and influence the costs.

As noted earlier, the PFR calculation can be considered relatively simple since its accuracy is affected by a smaller number of parameters. As can be seen in Table 5, there is a certain convergence in the results. This suggests that the CDW methodology, with its relative simplicity, can be used at the stage of the preliminary assessment of decisions made. Nevertheless, the calculation of the amount of air showed quite large differences. The dimensions of the PFR can be adopted as follows: the number of batteries is two; number of trains per battery is three; W × L × H (m) = 4 × 36 × 4. The overall volume is 3456 m^3^.

### 3.2. Modifying of the Scheme of Treatment

As already noted in various studies, the rate of wastewater disposal has decreased significantly recently [2,16]. Currently, in the Russian Federation, the typical daily SRWP at a level of 180 L per capita is admitted for calculations. Then, if the population of the considered city (50,000) does not change, the average daily flow of wastewater will be 9000 m^3^/day.

To determine the composition of wastewater during the modernization of treatment facilities, it is recommended to use the results of the analysis of wastewater samples taken. However, this information may not always be available, and in this case, it is allowed to determine the concentrations of pollutants by calculation (Equation (1)).

Table 6 provides information on the indicators of wastewater pollution that were used in the calculation of new facilities and in the design justification of WWTP modernization. A slight change in the norms for the mass of pollution per capita was fixed in the regulatory documents. As can be seen, the amount of pollution has increased significantly, while the requirements for wastewater treatment have become stricter. As a result, it requires the removal of not only organic contaminants but also nutrients, which was impossible to implement when using a PFR.

**Figure 4:**
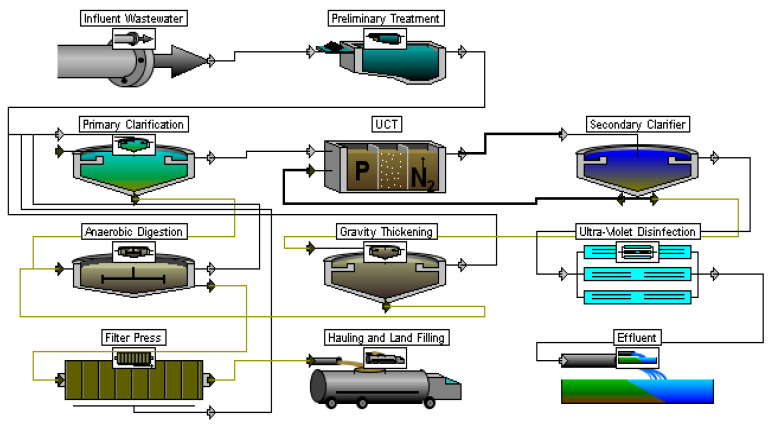
Proposed scheme for WWTP modernization (UCT-SC).

As part of the design justification for the modernization, the SoT should be considered, which allows the removal of a complex of contaminants. One such scheme is the technology of the University of Cape Town (UCT), whose application is aimed at removing both nitrogen and phosphorus through biological processes. In addition to the introduction of the UCT, the entire SoT has also been enhanced. The chlorine disinfection stage can normally be replaced with the UV unit. When processing excessive (or waste) activated sludge, as a rule, mechanical dewatering technologies are introduced instead of drying in natural conditions. The proposed wastewater treatment scheme is shown in Figure 4.

The calculation method [29] cannot be applied to the SoT, which provides the removal of carbon and nutrients, such as BNR. For manual calculation, a method [21] will be applied. An automated calculation using the CDW environment will also be performed. The calculation will be carried out at MLSS = 3 g/L, as well as for the PFR. The calculation results are presented in Table 7.

Based on the results of the analysis of the results obtained, the following conclusions can be drawn:

1. The application of nutrient removal technology requires a significant increase in volume, even despite the reduction in the flow of incoming wastewater;
2. The results of the calculation of the total volume of the structures showed a sufficiently high convergence, even despite the fundamental change (complication) of the cleaning technology;
3. With manual calculation, the volume of the anaerobic zone turned out to be significantly less (about three times) than with the automated calculation. The volumes of the anoxic zones coincided; in the case of manual calculation, the aerobic zone was obtained by about 12% more;
4. The calculation of the amount of air, as before, showed significant differences.

For this calculation case, it can be seen that the required volume of the structures has tripled, thus ensuring the necessary effect of wastewater treatment will require a significant expansion of the volume of the existing tanks. In the conditions of modernization and the fixed territory of treatment facilities, the expansion of the area is not always feasible.

### 3.3. MBR Application for Upgrading

The results of the calculations presented in Section 3.2 require further searching for optimal solutions in the field of WWTP upgrading. An increase in the required volume by three times cannot be called the most effective solution. Therefore, it is necessary to study solutions that would contribute to a reduction in volumes with the same treatment efficiency, which, in fact, means increasing the load on the facilities. Activated sludge plays a key role in the decomposition of pollutants; therefore, an increase in its amount in the reactor (the MLSS) may force the oxidation of contaminants in the reactor. However, there are also limiting factors for increasing the amount of activated sludge. If SCs are used to separate the mixed liquor into water and sludge, higher sludge concentrations in the activated sludge reactors may lead to lower efficiency of the SC operation. Membrane separation is an alternative to the gravitational separation of water and sludge. In wastewater treatment processes, membrane bioreactors (MBRs), which means an integrated system of membrane units and ASRs, are most common for these purposes [32]. The high efficiency of membrane sludge separation, almost regardless of its sedimentation properties, provides a TSS concentration in the effluent close to zero. In design practice, the TSS is usually indicated at less than 3 mg/l. Since activated sludge is also characterized by the BOD_5_ and nutrients, the operation of the MBR has a significant effect on reducing the values of these indicators. With proper mechanical strength of the membranes and qualified operation, the MBR can provide high reliability and efficiency for the entire stage of biological treatment in the majority of operational situations associated with discharges of pollutants, the deterioration of sludge properties, etc.

**Figure 5:**
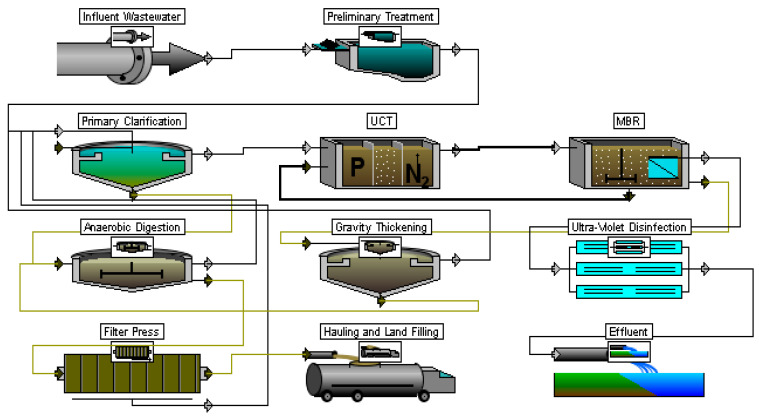
UCT-MBR scheme of treatment.

One of the advantages of MBR applications is the reduction of the required volume (and area) of the WWTPs. This is the result of an increase in MLSS in the ASR, which, in turn, significantly increases the load on the bioreactor. For the previous calculation, the value MLSS = 3 g/L was used, which is typical for ASRs with further gravity separation of water and activated sludge in the SC. In the case of MBRs, the optimal MLSS range is 7–12 g/L, which is 2–4 times higher. Due to the high value of MLSS in MBRs, it becomes possible not only to make treatment facilities more compact but also to ensure high-quality pollution removal from wastewater. In addition, high stability of the WWTP operation is achieved, which is important in the case of highly concentrated wastewater. A modified SoT (an MBR instead of an SC) is shown in Figure 5.

Table 8 shows the results of the calculation for a bioreactor’s (and the zones within it) volumes if the MLSS is in the range from 7 to 12 g/L with a step of 1 g/L in the manual and automated modes. It is seen that the results are most comparable when the MLSS is 9–12 g/L, and the difference between the tank’s volumes does not exceed 10%. It is interesting that within the growth of the MLSS, the reduction of volumes goes faster for the CDW method, mostly because of the aeration zone (the biggest among others). On the other hand, according to the MC method, the anaerobic zone has a similar size for all the values of the MLSS, as it depends only on the pollutant (P in particular) concentration but not the alterations of flow or the MLSS. Overall, the results are still looking similar (as for the PFR).

**Figure 6:**
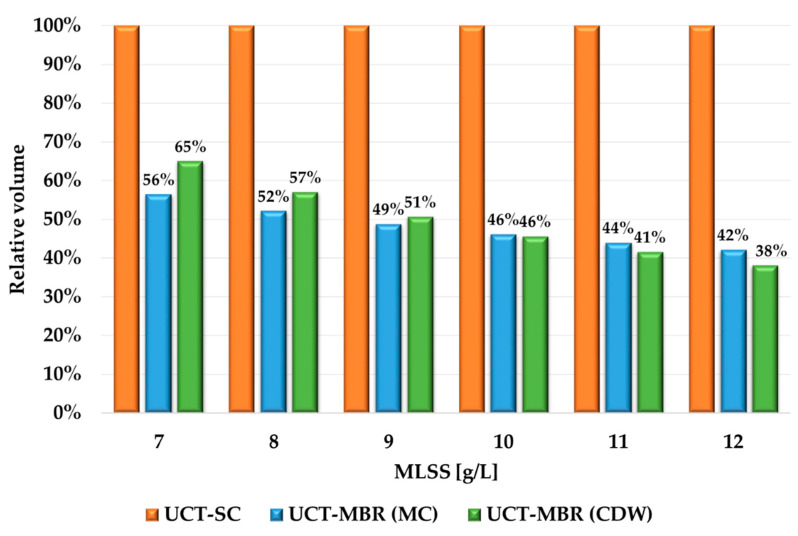
Visual presentation of ASR volume change after transition to membrane sludge separation.

Figure 6 shows the difference in the volumes, which was provided by the MLSS increase. As can be seen, when the MLSS grew from 3 g/L to 7 g/L, the required volume of the ASR with UCT-MBR technology is 35–45% less (depending on the calculation method) than for the UCT-SC scheme. When the MLSS are 12 g/L, the volume difference reaches 60%. What is also important from the viewpoint of our research is that both calculation methods showed comparable results. Thus, more variables had a low impact on the results of the calculation.

Another point of interest that should be highlighted is air consumption. As can be seen, the CDW method gives a constant value of air consumption with no dependence on the MLSS. Meanwhile, when the MLSS are 7–10 g/L, the MC method shows a slight reduction in air consumption, as it depends on the aeration intensity, which in turn depends on the aerobic zone’s HRT. If the calculated intensity is less than the required value, the required value should be used instead. When the MLSS ≥ 10 g/L, the calculated intensity is higher than the required value, and the air consumption remains constant. Supposedly, the MC approach seems more flexible, while the CDW procedure does not look clear enough. In this regard, further calculations of air consumption will be made only on an MC basis.

**Figure 7:**
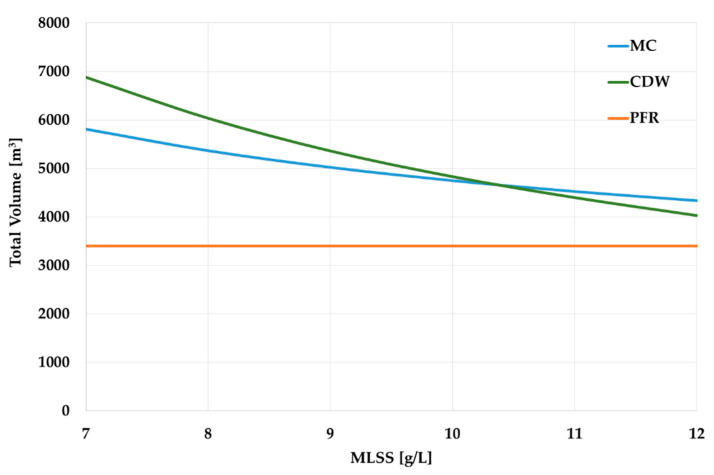
ASR volumes comparison.

If we compare the volumes of the PFR (the SoT before modernization) and the ASR in the UCT-MBR scheme, then, regardless of the calculation method and regardless of the MLSS for the UCT-MBR technology, the volume of the ASR (UCT-MBR) still exceeds the initial volume of the PFR (Figure 7). That means that an additional tank volume is required.

**Figure 8:**
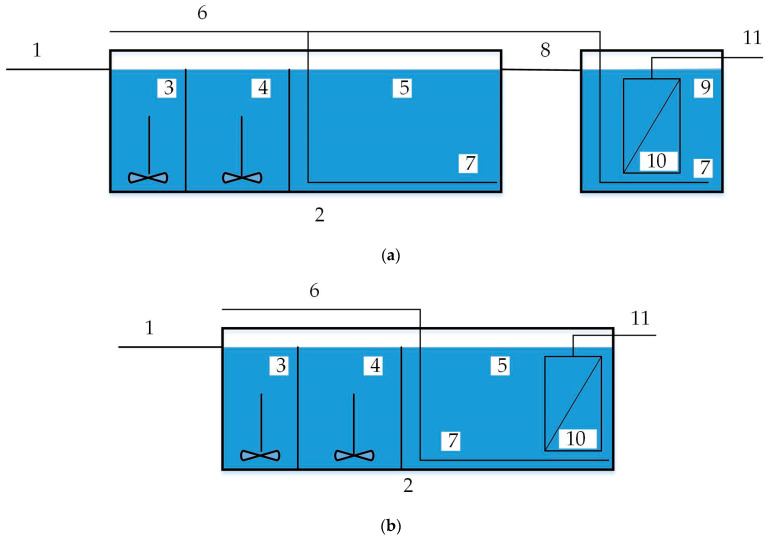
Simplified scheme of MBR with separate MR (**a**) and membrane units embedded in ASR (**b**): 1—influent form of primary clarifier; 2—ASR; 3—anaerobic zone; 4—anoxic zone; 5—aerobic zone; 6—air supply; 7—aeration system; 8—effluent to MR; 9—MR; 10—membrane unit; 11—permeate pumping.

However, the comparison in Figure 7 considers only the volumes of an ASR without the volume of the SC, which is obligatory for the PFR and UCT-SC sequence and which may require a significant volume, as the HRT is about 1.5–3 h. In the UCT-MBR scheme, a membrane reactor (MR) is required to place membrane units. The MR can be constructed as a separate tank. This solution allows easier maintenance of the membranes but requires additional construction (Figure 8a). On the other hand, the MR can be placed in the final part of the ASR, and no additional tank is needed (Figure 8b).

Therefore, it would be more correct to compare the total volumes for the ST stage; that is, an ASR + SC for the PFR technology and an ASR + MR for the UCT-MBR. Since the focus of the research is on the modernization of WWTPs, the more complicated option will be taken into account, which requires additional tanks and, of course, additional area for their location. To estimate the MR volume, first, the overall area of the membranes is needed. Since the area of the membranes depends primarily on the flow rate, regardless of the MLSS value, the area of the membranes may be taken as constant. According to both calculation methods, the membrane area should be approximately 30,000 m^2^ (*F_membr_*). The final value can be determined according to the size and number of the membrane units.

For the calculation, a membrane unit with a length, width, and height of 1.55 × 2× 2.6 m and an area (*F_unit_*) of the installed membranes of 1600 m^2^ will be used.

A number of units are required:

$$n_{m.unit}=\frac{F_{membr}}{F_{unit}}=\frac{30,000}{1600}=18.75\approx 19$$

The overall number of 20 units will be accepted for further calculations, with an area of 32,000 m^2^ and a total volume of 161 m^3^. The volume of the membrane reactor required for the placement of all the units, taking into account the space between them, is 400 m^3^.

**Figure 9:**
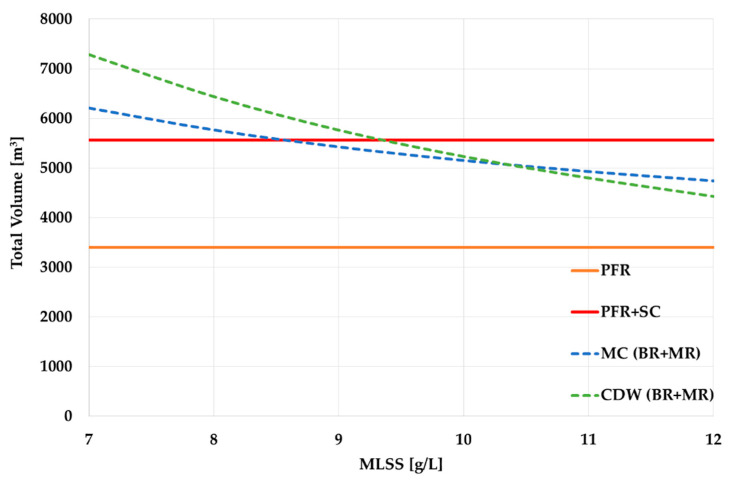
ASR and SC/MR volumes comparison.

A comparison (Figure 9) reveals that when the MLSS are 10–12 g/L, the required volume of the ASR + MR for UCT-MBR technology is now less than the ASR + SC volume for the PFR technology. If we consider these values from the point of view of modernization, it means that if UCT-MBR technology is applied, the WWTP upgrade would not require constructing new tanks. Nevertheless, existing tanks will certainly need repairing and/or re-equipment due to wear.

### 3.4. Cost Estimate

The next parameter to be considered within the framework of the calculation justification of WWTP modernization options is cost characteristics since the transition to the use of membranes requires significant costs. First, it is necessary to invest in the purchase of membranes. The unit cost may vary depending on the specific manufacturer. The current prices have a strong trend of reduction [33]. The actual level is about 4.5 EUR/m^2^ for membrane units only and 13.5 EUR/m^2^ for membranes plus the necessary equipment. Thus, the total cost (*C_membr_*) may reach 432,000 EUR. This can be attributed to capital costs. However, these costs may also be considered operational since the membranes’ lifetime (*LT_membr_*) is normally about 7–10 years, after which replacement is needed [33,34]. Taking into account the cost of chemical reagents (usually about 5% of the cost of the membranes) required for cleaning the membranes, the total cost will be about 455,000 EUR.

If investments are distributed throughout the lifetime, the specific costs of the membranes may be presented as:

$$S_{membr}=\frac{C_{membr}}{{LT}_{membr}\times Q_{daily}\times 365}=\frac{455,000}{10\times 9000\times 365}=0.014\frac{EUR}{m^{3}}$$

The next point to compare is operational costs, the major part of which is the air supply to the ASR. The cost calculation will be carried out manually since such calculations in the CDW software package are difficult due to the limited set of equipment in the software library.

The major part of the electricity costs is the energy consumption for aeration, which should be compared before and after the modernization. Prior to the upgrade, the necessary air supply to the PFR was approximately 6000 m^3^/h. As a rule, 2–4 blowers (*n_drive_*) are arranged in the WWTP. The most flexible controllability of the treatment facilities can be provided with the installation of four blowers. Then, the productivity of each should be at least 1500 m^3^/h. For normal operation, the blowers are equipped with electric motors with a capacity of about 22 kW (*P_drive_*).

Thus, the annual cost of electricity for the operation of the blowers (the PFR) will be:

$$P_{year}=P_{drive}\times n_{drive}\times 24\times 365=22\times 4\times 24\times 365=770,880\frac{kWh}{year}$$

The specific energy consumption is:

$${SC}_{air.PFR}=\frac{P_{drive}\times n_{drive}\times 24}{Q_{daily}}=\frac{22\times 4\times 24}{17,500}=0.12\frac{kWh}{m^{3}}$$

The obtained values are generally consistent with the previous studies [33,34,35].

Blowers in the UCT-SC scheme should support 5335 m^3^/h, which means that four existing ones should still be in action with the same value of *P_year_* as for the PFR. However, the specific energy consumption will change:

$${SC}_{air.UCT}=\frac{P_{drive}\times n_{drive}\times 24}{Q_{daily}}=\frac{22\times 4\times 24}{9000}=0.235\frac{kWh}{m^{3}}$$

If the UCT-MBR scheme is applied, the estimated air supply to the ASR value is reduced to 2352 m^3^/h, respectively; two of four blowers will be required to provide the necessary air supply.

Then, the annual cost of electricity for the operation of the blowers (aeration in the ASR) will be:

$$P_{year.UCT-M}=P_{drive}\cdot n_{drive}\times 24\times 365=22\times 2\times 24\times 365=385,440\frac{kWh}{year}$$

The specific energy consumption, in the meanwhile, will not change:

$${SC}_{air.UCT-M}=\frac{P_{drive}\times n_{drive}\times 24}{Q_{daily}}=\frac{22\times 2\times 24}{9000}=0.12\frac{kWh}{m^{3}}$$

In addition, it is separately required to provide physical cleaning of the membranes by aerating their surface. So, the air consumption for the aeration of the membranes of the adopted design should be estimated. Each membrane unit consists of 32 modules. The approximate air consumption for the aeration of each module is 6–7 m^3^/h. Then, the aeration of all the blocks will require 3840–4480 m^3^/h, which is 1.6–1.9 times higher than the cost of aeration in a bioreactor.

It should be taken into account that it is optimal to provide air supply for the aeration of the sludge mixture in the ASR and the aeration of the membranes from different groups of blowers [21]. Thus, the blowers for the ASR will be independent from those for the membranes, and vice versa. Since two blowers are now idle and do not supply air to the ASR, it is possible to switch them to supply air to the MR. However, their general air production (3000 m^3^/h < 3840–4480 m^3^/h) will not be sufficient, and a third additional blower (of the same performance) is required. Then, the annual cost of electricity for the operation of the blowers (for aeration in the MR) will be:

$$P_{year.UCT-M}=P_{drive}\times n_{drive}\times 24\times 365=22\times 3\times 24\times 365=578,160\frac{kWh}{year}$$

Specific energy consumption in the MR can now be determined:

$${SC}_{air.MR}=\frac{P_{drive}\times n_{drive}\times 24}{Q_{daily}}=\frac{22\times 3\times 24}{9000}=0.18\frac{kWh}{m^{3}}$$

The overall energy consumption for aeration in the ASR and MR will be:

$$P_{year.MBR}=P_{year.UCT-M}+P_{year.MR}=385,440+578,160=963,600\frac{kWh}{year}$$

The specific energy consumption for aeration is:

$${SC}_{air.MBR}={SC}_{air.BR}+{SC}_{air.MR}=0.12+0.18=0.3\frac{kWh}{m^{3}}$$

It is also possible to compare the costs of membrane acquisition and energy consumption for aeration. The price of 1 kWh is different in various regions. For instance, the average price in the EU at the end of 2022 was 0.25 EUR/kWh. So, if we compare the previously calculated *S_membr_* with this value, the specific cost of the membrane will be equal to the specific energy consumption of 0.04 kWh/m^3^. In the case of Russia, with the average price of 1 kWh equal to 0.08 EUR, the specific cost of the membrane will be equal to the specific energy consumption of 0.11 kWh/m^3^.

**Figure 10:**
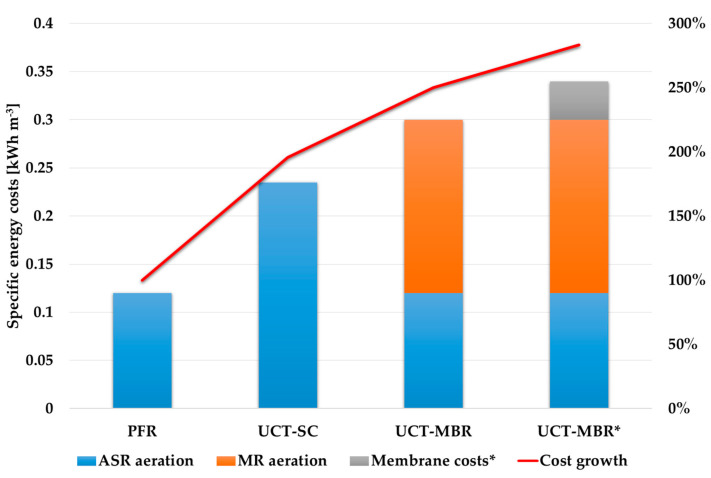
Comparison of specific energy costs.

Figure 10 shows calculated values of the specific energy consumption for the considered technological schemes. The UCT-MBR* bar shows the overall specific energy consumption if membrane costs are presented as specific energy consumption. It can be seen that the transition to an advanced scheme of treatment leads to a significant growth in energy consumption [16,33]. The application of an MBR means less tank volume for the ASR, which reduces the energy consumption for aeration in the ASR; however, membrane aeration is an extremely energy-consuming process. If we consider membrane costs throughout their life cycle (10 years), they account for about 13% of the total cost of electricity for aeration within this period.

## 4. Conclusions

Based on the results of the calculations carried out (including using the CapdetWorks 4.0 software package), the following conclusions can be drawn:

1. The comparison of the calculation methods showed convergence in the calculated volumes of the treatment plant tanks. However, related characteristics, such as airflow, require additional clarification;
2. During the modernization of WWTPs, the change in the concentration of pollutants was the determining factor in calculating the volume of the facilities;
3. With the same flow rate of incoming wastewater and the same concentration of pollutants, the use of membrane bioreactors can reduce the required volumes of the activated sludge reactors by 1.5–3 times, depending on the MLSS value;
4. The calculations showed that in the considered upgrade case (the transition from a PFR to a UCT-MBR within MLSS = 10 g/L), additional tanks for secondary treatment are not required. Specific energy costs for aeration in both cases are equal to 0.12 kWh/m^3^. However, the application of the membranes requires significant costs for their aeration, which reach almost 150% of the aeration costs in the ASR. In that case, the overall specific energy cost for aeration will be approximately 0.3 kWh/m^3^;
5. A comparison of energy costs for aeration and membrane costs (which were distributed through the lifetime of 10 years) showed that membrane costs were approximately 10–15% of the overall energy costs for aeration. However, this value may be different in various regions, depending on energy prices.

## Figures and Tables

**Table 1:** Distribution of daily wastewater influent (L per capita) [15].

Flow Range	WWTPs According to Their Capacity [%]		
	>300 k m^3^/Day	100–300 k m^3^/Day	<100 k m^3^/Day
Mean values (70% of overall)	270–440	180–400	180–410
Minimum	240	130	80
Maximum	550	670	710

**Table 2:** Distribution of TSS/BOD_5_ pollution at WWTPs [15].

Range of Pollutants Concentration TSS/BOD_5_ [mg/L]	Pollutants Distribution within WWTPs Capacity [%]		
	>300 k m^3^/Day	100–300 k m^3^/Day	<100 k m^3^/Day
Low-concentrated (<150/<130)	21/22	28/31	30/30
Medium-concentrated (150–250/150–230)	47/42	48/48	50/57
Highly concentrated (>250/>230)	32/36	24/21	20/13

**Table 3:** Distribution of N/P pollution at WWTPs [15].

Range of Pollutants Concentration N/P [mg/L]	Pollutants Distribution within WWTPs Capacity [%]		
	>300 k m^3^/Day	100–300 k m^3^/Day	<100 k m^3^/Day
Low-concentrated (<25/<2.2)	5/22	41/33	30/30
Medium-concentrated (25–35/2.2–3)	67/39	40/41	38/33
Highly concentrated (>35/>3)	28/39	19/26	32/37

**Table 4:** Specific values of pollutants amount per capita and initial pollutant concentrations prior to modernization.

Indicator	Amount of Pollutants Per Capita, *A_PC_* [g/Day] [29]	Pollutant Concentration [mg/L]	Limits in Action before 2000 [mg/L]
TSS	65	186	10–15
BOD_5_ of untreated wastewater	75	214	10–15
Chemical oxygen demand (COD)	n/a	n/a	n/a
Ammonia (NH_4_)	8	23	n/a
Phosphates (PO_4_)	3.3	9.4	n/a

n/a—not available.

**Table 5:** Calculated PFR parameters.

Parameters	MC [29]	CDW Method [30]
Volume of PFR [m^3^]	3403	3460
HRT [h]	4.62	4.7
Air flow [m^3^/h]	5968	4130
Volume of SC [m^3^]	2160	1950

**Table 6:** Specific values of pollutants amount per capita and initial pollutant concentrations after modernization.

Indicator	Amount of Pollutants Per Capita, *A_PC_* [g/Day] [31]	Pollutants Concentration [mg/L]	Current Limits in Action [mg/L]
TSS	65	372	10
BOD_5_ of untreated water	60	333	2.1
Chemical oxygen demand (COD)	120	666	n/a
Ammonia (NH_4_)	8.8	49	0.4
Phosphates (PO_4_**)**	1	9.4	0.2

n/a—not available.

**Table 7:** Calculation results for UCT-SC scheme.

Parameters	MC	CDW
Total Volume of ASR [m^3^]	10,307	10,640
Volume of Anaerobic Zone [m^3^]	528	1600
Volume of Anoxic Zone [m^3^]	3725	3720
Volume of Aerobic Zone [m^3^]	6054	5320
Total HRT [h]	26.3	27.16
Air Flow [m^3^/h]	5335	3710

**Table 8:** Calculation results for UCT-MBR.

MLSS [g/L]	Volume [m^3^]								Total HRT [h]		Air Flow [m^3^/h]	
	Total		Anaer. Zone		Anoxic Zone		Aer. Zone					
	MC	CDW	MC	CDW	MC	CDW	MC	CDW	MC	CDW	MC	CDW
7	5811	6880	539	1030	2008	2410	3263	3450	15.05	17.81	2855	4260
8	5363	6035	539	905	1840	2110	2990	3090	13.9	15.62	2615	4260
9	5025	5360	539	804	1709	1880	2777	2680	13.0	13.87	2429	4260
10	4749	4830	539	724	1604	1690	2606	2410	12.3	12.5	2385	4260
11	4524	4400	539	658	1518	1540	2467	2190	11.72	11.4	2385	4260
12	4337	4023	539	603	1446	1410	2351	2010	11.23	10.41	2385	4260

## Data Availability

Not applicable.
